# Combining Ability of Pod Yield and Related Traits of Groundnut (*Arachis hypogaea* L.) under Salinity Stress

**DOI:** 10.1155/2014/589586

**Published:** 2014-03-09

**Authors:** Md. Abul Kalam Azad, Md. Shah-E-Alam, Md. Abdul Hamid, Mohd Y. Rafii, M. A. Malek

**Affiliations:** ^1^Plant Breeding Division, Bangladesh Institute of Nuclear Agriculture, Mymensingh 2202, Bangladesh; ^2^Department of Genetics and Plant Breeding, Bangladesh Agricultural University, Mymensingh 2202, Bangladesh; ^3^FormerDirector General, Bangladesh Institute of Nuclear Agriculture, BAU Campus, Mymensingh 2202, Bangladesh; ^4^Institute of Tropical Agriculture, Universiti Putra Malaysia (UPM), 43400 Serdang, Selangor, Malaysia

## Abstract

A study was performed using 6 × 6 F_1_ diallel population without reciprocals to assess the mode of inheritance of pod yield and related traits in groundnut with imposed salinity stress. Heterosis was found for pod number and yield. Data on general and specific combining ability (gca and sca) indicated additive and nonadditive gene actions. The gca: sca ratios were much less than unity suggesting predominant role of nonadditive gene effects. Cultivars “Binachinabadam-2” and “Dacca-1” and mutant M_6_/25/64-82 had the highest, second highest, and third highest pod number, as well as gca values, respectively. These two cultivars and another mutant M_6_/15/70-19 also had the highest, second highest, and third highest pod yield, as well as gca values, respectively. Therefore, “Dacca-1”, “Binachinabadam-2”, M_6_/25/64-82, and M_6_/15/70-19 could be used as source of salinity tolerance. Cross combinations showing high sca effects arising from parents with high and low gca values for any trait indicate the influence of nonadditive genes on their expression. Parents of these crosses can be used for biparental mating or reciprocal recurrent selection for developing high yielding varieties. Crosses with high sca effects having both parents with good gca effects could be exploited by pedigree breeding to get transgressive segregants.

## 1. Introduction 

Salinity is one of the most serious environmental factors limiting crop productivity worldwide [1]. This stress is complex and causes a number of detrimental effects: (i) reduces the ability of plants to absorb water, called water, or osmotic stress, (ii) causes ionic imbalance, (iii) imposes hyperosmotic shock by decreasing chemical activity of water and causing loss of cell turgor, and (iv) reduces chloroplast stromal volume and generates reactive oxygen species (ROS). Globally, nearly 100 million ha of land is affected by salinity which accounts for 6-7% of the total arable land [2]. In Bangladesh, in the coastal belt, 1.02 million ha of cultivated land is affected by varying degrees of soil salinity, 2.0 to >16 dS/m [3, 4]. Soil salinity in these areas remains lower from June to November but increases and accentuates from May onwards [5].

Cultivation of high water demanding crop is not possible during the dry period (November to May) in the saline areas of Bangladesh because lack of suitable irrigation water. Groundnut (*Arachis hypogaea* L.) requires only 350 mm water to complete its life cycle [6] and is mostly grown under rainfed conditions during November to April/May. The climatic and edaphic conditions of saline areas of Bangladesh are suitable for growing groundnut, but this is limited by soil salinity and high pH [4, 7]. In order to grow in these conditions, a groundnut cultivar needs to tolerate up to 8 dS/m salinity. If this was possible the groundnut area could be expanded to an additional 0.596 million hectares of saline-affected soil.

In order to design an appropriate selection strategy for the trait(s) of interest, it is imperative to assess the mode of its inheritance, especially with respect to being additive or nonadditive. Diallel analysis [8, 9] helps to identify parents with additive and nonadditive effects for specific traits that may be used in breeding [10]. Prediction of genetic diversity and general combining ability (gca) of parents before crossing reduces the number of crosses and progenies to be screened which reduces cost and time [11]. No data are available for groundnut till under salinity stress condition, and therefore, this study was set up to (i) elucidate the mode of inheritance of genes governing the expression of pod yield and related traits under 8 dS/m salinity stress, (ii) select parents for hybridization programs in developing salt tolerant groundnut cultivars, and (iii) assess good specific combining parental lines.

## 2. Materials and Methods

### 2.1. Materials

A 6 × 6 F_1_ diallel population without reciprocal was used in this study. The parents of this population were discriminated as being salt tolerant/sensitive based on pod number and yield under salt stress [12]. Four salt tolerant mutants/cultivars: M_6_/15/70-19, “Binachinabadam-1”, M_6_/25/64-82, and “Binachinabadam-2” and 2 sensitive cultivars: “Dacca-1” and “Zhingabadam” were crossed following the method of Kumar and Patel [13] with some modifications. The source, origin, method of development, and botanical type of the parents are shown in Table 1. It is important to mention here that the parent of “Binachinabadam-1”, “Binachinabadam-2”, M_6_/15/70-19, and M_6_/25/64-82 was Mut-6. Mutant-6 was derived by irradiating the seeds of “Dacca-1”, a local land race; thus, “Dacca-1” is the common parent of “Binachinabadam-1”, “Binachinabadam-2”, M_6_/15/70-19, and M_6_/25/64-82.

### 2.2. Experimental Site, Experiment Design, and Plant Culture

The experiment was conducted in the glasshouse of Bangladesh Institute of Nuclear Agriculture (BINA), Mymensingh, with temperature and relative humidity ranges of 25–35°C and 70–90%, respectively. The study was set up as a completely randomized design with four replications, under 8 dS/m salinity level, imposed during flowering till harvest stages as flowering stage is the most sensitive stage to salinity stress [14]. Five pregerminated seeds of each parent and cross were sown on February 11, 2007 in earthen pots of size 27 × 22 cm, lined with polyethylene sheet and filled with 8.0 kg soil mixture, prepared with sandy loam soil and well-rotted farm yard manure in a 1 : 1 ratio. The fertilizer needed for each pot was determined following the Fertilizer Recommendation Guide-2005 [15]. The total amount of nitrogen, phosphorus, potassium, sulphur, and zinc was applied in the form of urea, triple super phosphate (TSP), muriate of potash (MP), gypsum, and zinc sulphate. These were mixed thoroughly with the soil in each pot before sowing. When the plants were established, only three healthy plants were kept in each pot. The pots were kept free from weeds and protected from insect pests and diseases by spraying appropriate pesticides as and when necessary.

### 2.3. Imposing Salinity and Data Recording

Saline water was synthesized by using a mixture of different salts: 50% NaCl, 15% Na_2_SO_4_, 10% NaHCO_3_, CaCl_2_, and MgCl_2_ together with 5% MgSO_4_ by weight to simulate saline ground water of saline areas of Bangladesh [4]. A stock solution was prepared by dissolving 50 grams of the salt mixture in 1 liter of tap water. The salinity of the stock solution was then adjusted to 80 dS/m. The total amount of stock solution needed to raise 8 dS/m salinity of the soil mixture was estimated with the following equation:

(1) $$\begin{array}{c} V_{1}S_{1}\;=\;V_{2}S_{2}, \end{array}$$

where

 
*V*_1_ = volume of soil mixture in a pot,
 
*S*_1_ = desired salinity - initial salinity of the soil,
 
*V*_2_ = volume of water at 70–80% plant available water (PAW),
 

*S*_2_ = salinity of stock solution.

Volume of soil mixture (*V*_1_) was determined using the following formula:

(2) $$\begin{array}{c} V_{1}=\frac{\text{Weight}\;\text{of}\;\text{oven}\;\text{dried}\;\text{soil}}{\text{Bulk}\;\text{density}\;\text{of}\;\text{soil}}. \end{array}$$

Volume of water (*V*_2_) was determined by dividing the weight of water by its density (0.98 g/cc).

The estimated amount of stock solution was then diluted to the desired salinity levels by adding tap water and then applied from flowering until harvest. The total amount of saline water for the respective treatments was applied in installments. At each installment, 0.5 to 1.0 liter saline water was applied so that the moisture content of the pots remained between 70 and 80% of PAW. For the control, the same amount of tap water was applied. All plants in a pot were uprooted at full maturity and washed with running tap water. Plant height, branch number, and pod number were recorded before sun drying. After sun drying, pod yield per plant was recorded. There were altogether 12 plants in each F_1_ populations, three plants in a pot and 4 such pots in each population.

### 2.4. Genetic Analysis

Combining ability analysis was performed following Method 2, Model 1 of Griffing [16]. ANOVA for combining ability analysis in Method 2 Model 1 is shown in Table 2.

### 2.5. Model Followed

The mathematical model for the combining ability analysis in model 1 was as follows:

(3) $$\begin{array}{c} Y_{ij}=m+g_{i}+g_{j}+s_{ij}+\;\frac{\text{l}}{bc}\sum \sum e_{ijkl}, \end{array}$$

where *i*, *j* = 1,…, *n* (*n* = number of parents),   *k* = 1,…, *b* (*b* = number of blocks/replications),  and  *l* = 1,…, *c* (*c* = number of observation in each plot).

*Y*_*ij*_ is the mean of *i* × *j*th genotype over *k* and *l*; *m* is the population mean; *g*_*i*_ is the gca effect of *i*th parent; *g*_*j*_ is the gca effect of *j*th parent. *s*_*ij*_ is the sca effect and 1/*bc*∑∑*e*_*ijkl*_ is the mean error effect.

Restrictions imposed are ∑_*i*_ *g*_*i*_ = 0 and ∑_*j*_ *s*_*ij*_ + *s*_*ii*_ = 0 (for each *i*).

The sum of squares (SS) were calculated as follows:

(4) SSg=1n+2[∑i(Yi.+  Yii)2−  4nY2..],SSs=∑∑  Yij2−1n+2∑(Yi.+Yii)2 +  2(n+1)(n+2)Y2..,

where SS_*g*_ is the sum of squares due to gca, SS_*s*_  is the sum of squares due to sca,  *Y*_*i*_ is the array total of the *i*th parent,  *Y*_*ii*_  is the mean value of the *i*th parent,  *Y*.. is the grand total of the 1/(2n(n − 1)) crosses and parental values,  and  *Y*_*ij*_ is the progeny mean values in the diallel table.

Thus the effects were calculated as follows:

(5) gi=1n+2[∑(Yi.+  Yii)−2nY..],sij=Yij  −1n+2[Yi.+  Yii  +  Y.j  +Yjj] +2(n+1)(n+2)Y..

Variance of effects was calculated as follows:

- Var⁡(*g*_*i*_) = (*n* − 1)*δ* ^2^_*e*_/*n*(*n* + 2),
- Var⁡(*s*_*ij*_) = 2(*n* − 1)*δ* ^2^_*e*_/(*n* + 1)(*n* + 2).

SE (standard error) was calculated as the square root of the variance.

## 3. Results

### 3.1. ANOVA and Mean Squares

Mean squares (ANOVA) for pod yield and some related traits of a 6 × 6 F_1_ half diallel population at 8 dS/m salinity stress are presented in Table 3. Results revealed highly significant (*P* ≤ 0.01) differences between genotypes for plant height, branch number, pod number, and pod yield and not significant difference for branch number. The parents showed such significant differences for plant height and pod yield but just significant and not significant differences for pod and branch number. Like genotype, the F_1_s showed highly significant differences for all the traits, but the term P versus F_1_ appeared highly significant for pod number and yield but not significant for all the other traits.

### 3.2. Means

Means of pod yield and some related traits of the parents at 8 dS/m salinity stress, imposed during flowering till harvesting stages, are presented in Table 4. In this experiment, “Zhingabadam” had the tallest height, appeared statistically identical with “Dacca-1” only. However, the mutant M_6_/15/70-19 had the shortest height showing significant difference with all others except “Binachinabadam-2”. Branch number did not show any significant difference amongst the parents. Pod number was the highest in “Binachinabadam-2”, and it differed significantly with that of all the remainders. In this experiment, only “Zhingabadam” failed to produce any pod. However, 3 of the 6 mutants/varieties did not have any mature pod, and thus they have no pod yield. Of the remaining three, “Binachinabadam-2” had significantly the highest pod yield followed by “Dacca-1”.

### 3.3. Combining Ability

Analysis of variance data for combining ability is presented in Table 5. Results exhibited highly significant differences for general combining ability (gca) and specific combining ability (sca) effects for all the traits except branch number. Branch number showed not significant difference for gca (data not shown). The sca components of variance were always higher than that of its gca counterpart.

### 3.4. General and Specific Combining Ability

Estimates of gca and sca effects of the tested genotypes are presented in Tables 6 and 7, respectively. The parent “Dacca-1” showed highly significant positive gca values for all the traits, while “Binachinabadam-2” showed highly significant and positive gca values for only pod number and yield. Like the tallest cultivar, “Dacca-1”, “Zhingabadam” had significant positive gca for plant height. In contrast, the shortest mutant, M_6_/15/70-19, had the lowest gca for plant height. The remaining genotypes had not significant negative gca values for plant height. The two genotypes with the highest pod number and yield (Table 4) also had highly significant positive gca values (Table 6). In contrast, all other genotypes had mostly highly significant negative (low) gca values, except M_6_/25/64-82 for pod number.

For plant height, the cross “Binachinabadam-1” × “Binachinabadam-2” appeared as the best specific combination followed by “Dacca-1” × M_6_/25/64-82, “Zhingabadam” × M_6_/15/70-19, and “Dacca-1” × “Binachinabadam-2” crosses (Table 7). These originated from crossing between parents having gca values: not significant negative versus not significant negative, highly significant positive versus not significant negative, significant positive versus highly significant negative, and highly significant positive versus not significant negative, respectively. In contrast, the cross “Dacca-1” × “Zhingabadam” had the worst sca effect followed by “Dacca-1” × “Binachinabadam-1”, “Zhingabadam” × “Binachinabadam-2”, “Zhingabadam” × “Binachinabadam-1”, “Dacca-1” × M_6_/15/70-19, and M_6_/25/64-82 × “Binachinabadam-2”. The parents of these crosses had highly significant versus just significant positive, significant positive versus not significant negative, significant positive versus not significant negative, highly significant positive versus negative, and not significant negative versus not significant negative gca values, respectively.

For pod number, 8 cross combinations showed highly significant positive sca with “Dacca-1” × M_6_/15/70-19 being the best followed by “Dacca-1” × “Binachinabadam-2”, M_6_/15/70-19 × “Binachinabadam-1”, “Zhingabadam” × “Binachinabadam-1”, “Zhingabadam” × M_6_/25/64-82, “Zhingabadam” × “Binachinabadam-2”, M_6_/25/64-82 × “Binachinabadam-2”, and “Zhingabadam” × M_6_/15/70-19 (Table 7). These crosses were obtained from crossing between parents with highly significant positive versus highly significant negative, highly significant positive versus highly significant positive, highly significant negative versus highly significant negative, highly significant negative versus highly significant negative, highly significant negative versus not significant positive, highly significant negative versus highly significant positive, not significant positive versus highly significant positive and highly significant negative versus highly significant negative gca values, respectively. In contrast, all the remaining crosses except “Dacca-1” × “Zhingabadam” showed highly to just significant negative sca with “Binachinabadam-1” × “Binachinabadam-2” being the lowest and were followed by M_6_/15/70-19 × “Binachinabadam-2”, “Dacca-1” × M_6_/25/64-82, “Binachinabadam-1” × “Binachinabadam-2”, M_6_/15/70-19 × M_6_/25/64-82, and “Dacca-1” × “Binachinabadam-1” in decreasing order. These crosses were obtained from crossing between parents with highly significant negative versus highly significant positive, highly significant negative versus highly significant positive, highly significant positive versus not significant positive, highly significant negative versus not significant positive, and highly significant positive versus highly significant negative gca values, respectively.

However, for pod yield, out of 15 cross combinations, 7 had shown heterotic performances. They were “Dacca-1” × M_6_/15/70-19, “Dacca-1” × “Binachinabadam-2”, “Zhingabadam” × M_6_/15/70-19, “Zhingabadam” × “Binachinabadam-1”_,_ “Zhingabadam” × M_6_/25/64-82_,_ M_6_/15/70-19 × “Binachinabadam-1”, and M_6_/25/64-82 × “Binachinabadam-2”.

## 4. Discussion 

Tolerance to salinity is highly influenced by the environmental factors [17]. The variety “Dacca-1” showed higher tolerance in terms of both pod number and yield when exposed to 7–9 dS/m salinity stresses from flowering till harvest stages in glasshouse condition from August to January by showing relative pod and kernel yields higher than even the unstressed treatment [18]. In contrast, the same variety when exposed to 8 dS/m salinity stress in same glass house condition from April to August could not produce any pod [12]. This was due to high temperature during the reproductive stage. Under glass house condition, sometimes the maximum temperature exceeded 50°C during the experimental period. The optimum air temperature during the day in groundnut ranges from 25°C to 30°C for vegetative growth and from 24°C to 28°C for reproductive growth and development [19, 20]. This explains well the fluctuation in tolerance of the variety “Dacca-1” in the two studies. In this study, the variety “Dacca-1” appeared tolerant as it produced the second highest pod number and yield. This was attributed to the optimum temperature during the reproductive period in the glass house. The variety “Binachinabadam-2” and the mutant M_6_/15/70-19 appeared tolerant in this study as these could produce pod yield (Table 4) and also in the screening study [12] in which temperature during the reproductive stage exceeded 50°C. This means that these two are most tolerant to 8dS/m salinity stress even under high temperature during the reproductive stage. Therefore, it is clear that in this study “Dacca-1”, “Binachinabadam-2” and M_6_/15/70-19 were found to be tolerant and the remainders were found to be sensitive. This may result in screening study [12].

A detailed genetic study is a prerequisite in determining the mode of inheritance of traits which ultimately helps adopt better planning and execution in a plant breeding improvement programme. This study was to assess this with respect to pod yield and some related traits in groundnut exposed to salt stress. The term parent versus offspring appeared significant for pod number and yield only but not significant difference for plant height and branch number (Table 3). This suggests presence of heterosis for pod number and yield but its absence for plant height and branch number.

The significant general and specific combining ability data (Table 5) conform to other publications [21–24] that investigated salinity tolerance in many different crops. Baker [25] suggested that general and specific combining ability should be assessed by estimating the components of variance, expressing as *σ* ^2^_*g*_/*σ* ^2^_*s*_ ratio. The closer the ratio to unity is, the greater would be the magnitude of additive genetic effects. The ratios computed in this study were much less than unity (Table 5) suggesting predominant role of nonadditive gene effects in their inheritance. This result is in agreement with that of many workers working with salinity tolerance in many different crops [26–29]. The higher values of sca than gca could be due to repulsion phase linkage and linkage disequilibrium [30]. Verma and Srivastava [31] observed high sca effects resulting from the dominance and interaction or epistatic effects that exist between the hybridizing parents. Additionally, higher sca than gca can be explained in other different ways: (i) negative associations between genes [30], (ii) previous selection that narrowed the genetic base of the lines tested [32], (iii) directional selection [33], and (iv) use of closely related parents [34]. In these studies, since the genotypes used had been selected mainly for high yield, this might have narrowed their genetic bases. Moreover, most of the genotypes tested have common origins, for example, the common parent “Dacca-1”, which could be a reason for narrow genetic base.

Two cultivars, “Binachinabadam-2”, and “Dacca-1” and mutant M_6_/25/64-82 with the highest, second highest, and third highest pod number, respectively, had also highest, second highest, and third highest gca values for pod number (Table 6). For pod yield, the above two cultivars and the mutant M_6_/15/70-19 had highest, second highest, and third highest gca values (Table 6). Obviously, these four materials: “Dacca-1”, “Binachinabadam-2”, M_6_/25/64-82, and M_6_/15/70-19 could be used as major source of salinity tolerance. The high sca effects for pod number were obtained by crossing parents with high × low, high × high, low × low, and low × high gca values (Table 6 and Table 7). In contrast, high sca effects for pod yield were obtained by crossing parents with either high × low or low × high gca values. The cross combinations showing high sca effects arising from parents with high and low gca values for any trait indicate that there is influence of nonadditive genes on their expression [35]. Parent of these crosses can be used for biparental mating or reciprocal recurrent selection for developing superior varieties with high yield. Crosses with high sca effects having both parents with good gca effects could be exploited by pedigree method of breeding to get transgressive segregants.

## Figures and Tables

**Table 1:** Origin/sources of the groundnut genotypes used in this experiment together with method of development.

SL. no.	Genotype	Botanical type	Origin	Status and method of development	Parent	Gamma-ray dose (KR)
1	“Dacca-1”	Spanish	BARI	Introduction	—	—
2	“Zhingabadam”	Valencia	BARI	Introduction	—	—
3	M_6_/15/70-19	Spanish	BINA	Mutant selected after mutation induction	Mut-6	15
4	“Binachinabadam-1”	Spanish	BINA	Variety developed after mutation	Mut-6	20
5	M_6_/25/64-82	Spanish	BINA	Line selected after mutation induction	“Dacca-1”	35
6	“Binachinabadam-2”	Spanish	BINA	Variety developed after mutation induction	Mut-6	25

BARI: Bangladesh Agricultural Research Institute.

**Table 2:** 

Source	df	SS	MS	Expected mean squares
gca	*p* − 1	SSg	Mg	*δ* ^2^ *e* + ((p + 2)/(p − 1))∑*g* ^2^i
sca	*p*(*p* − 1)/2	SSs	Ms	*δ* ^2^ *e* + (2/*p*(*p* − 1))∑∑*s* ^2^ *ij*
Error	(*b* − 1)(*e* − 1)	SSe	Mé	*δ* ^2^ *e*

Note: *p* stands for number of parents.

**Table 3:** Analysis of variance of pod yield and related traits in a 6 × 6  F_1_ diallel population at 8 dS/m salinity imposed during flowering till harvest stages.

Sources of variation	df	Plant height	Branch number	^ ++^Pod number	^ ++^Pod yield
Genotype	20	55.88**	0.63**	0.85**	0.45**
Parent (P)	5	109.13**	0.06	1.41*	0.71**
F_1_ *s*	14	40.81**	0.83**	0.61**	0.35**
P versus F_1_	1	0.70	0.63	1.34**	0.67**
Error	42	3.42	0.20	0.01	0.01

∗∗ indicates significant at *P* ≤ 0.05; ++ indicates square-root transformed data used.

**Table 4:** Means of pod yield and related traits of the parents as influenced by 8 dS/m salinity imposed during flowering till harvest stages.

Parents	Plant height (cm)	Branch/plant (no.)	^ ++^Pod/plant (no.)	^ ++^Pod yield/plant (g)
“Dacca-1”	23.83	3.89	2.33	1.53
“Zhingabadam”	24.50	3.84	0.71	0.71
M_6_/15/70-19	10.17	4.00	1.41	1.06
“Binachinabadam-1”	17.25	4.17	1.79	0.71
M_6_/25/64-82	13.33	4.00	2.26	0.71
“Binachinabadam-2”	12.67	3.78	2.51	1.86
LSD_0.05_	3.01	NS	0.01	0.01

NS: nonsignificant; ++: square-root transformed data.

**Table 5:** Analysis of variance for combining ability of pod yield and related traits in a 6 × 6  F_1_ diallel population subject to salt stress.

Item	df	Plant height	^ ++^Pod number	^ ++^Pod yield
gca	5	26.13**	0.64**	0.20**
sca	15	16.13**	0.16**	0.13**
Error	42	1.14	0.003	0.003

Variance components				
gca		3.12	0.08	0.02
sca		14.98	0.16	0.13
gca : sca		0.21	0.49	0.19

∗∗ indicates significance at *P* ≤ 0.05; ++ indicates square-root transformed data used.

**Table 6:** Estimates of gca effects of parental lines in a 6 × 6  F_1_ diallel population for pod yield and related traits during flowering till harvest stages under 8 dS/m salinity.

Parents	Plant height	^ ++^Pod number	^ ++^Pod yield
“Dacca-1”	2.63**	0.38**	0.15**
“Zhingabadam”	1.42*	−0.41**	−0.06*
M_6_/15/70-19	−2.50**	−0.11**	0.09**
“Binachinabadam-1”	−0.04	−0.14**	−0.19**
M_6_/25/64-82	−0.77	0.05	−0.17**
“Binachinabadam-2”	−0.74	0.23**	0.17**
SE	0.34	0.02	0.02

∗∗ and ∗ indicate significance at *P* ≤ 0.05 and *P* ≤ 0.01, respectively; ++ indicates square-root transformed data used.

**Table 7:** Estimates of sca effects in a 6 × 6  F_1_ diallel population of pod yield and related traits after salt stress.

Cross combinations	Plant height	^ ++^Pod number	^ ++^Pod yield
“Dacca-1” × “Zhingabadam”	−6.62**	0.10*	−0.07*
“Dacca-1” × M_6_/15/70-19	−1.58*	0.66**	0.20**
“Dacca-1” × “Binachinabadam-1”	−4.61**	−0.05	−0.01
“Dacca-1” × M_6_/25/64-82	5.34**	−0.24**	−0.17**
“Dacca-1” × “Binachinabadam-2”	3.91**	0.52**	0.12**
“Zhingabadam” × M_6_/15/70-19	3.96**	0.19**	0.18**
“Zhingabadam” × “Binachinabadam-1”	−2.67**	0.30**	0.28**
“Zhingabadam” × M_6_/25/64-82	−0.19	0.25**	0.51**
“Zhingabadam” × “Binachinabadam-2”	−4.22**	0.24**	−0.04
M_6_/15/70-19 × “Binachinabadam-1”	0.03	0.49**	0.75**
M_6_/15/70-19 × M_6_/25/64-82	0.14	−0.10*	−0.10**
M_6_/15/70-19 × “Binachinabadam-2”	0.12	−0.28**	−0.44**
“Binachinabadam-1” × M_6_/25/64-82	−0.65	−0.16**	−0.25**
“Binachinabadam-1” × “Binachinabadam-2”	6.83**	−0.57**	−0.42**
M_6_/25/64-82 × “Binachinabadam-2”	−1.37*	0.12**	0.31**
SE	0.45	0.02	0.02

∗ and ∗∗ indicate significance at *P* ≤ 0.05 and *P* ≤ 0.01, respectively; ++ indicates square-root transformed data used.
